# Secondhand smoke (SHS) exposure at home and at the workplace among non-smokers in Malaysia: Findings from the Global Adult Tobacco Survey 2011

**DOI:** 10.18332/tid/95188

**Published:** 2018-10-24

**Authors:** Kuang Hock Lim, Hui Li Lim, Chien Huey Teh, Chee Cheong Kee, Pei Pei Heng, Yong Kang Cheah, Sumarni Mohd Ghazali

**Affiliations:** 1 Institute for Medical Research, Kuala Lumpur, Malaysia; 2 Hospital Sultan Haji Ahmad Shah, Pahang, Malaysia; 3 University Utara Malaysia, Sintok, Kedah, Malaysia

**Keywords:** secondhand smoke, smoke-free home, non-smoking workplace, non-smoker adults

## Abstract

**INTRODUCTION:**

Understanding the prevalence of secondhand smoke (SHS) exposure and the associated factors is beneficial for the formulation of effective measures to reduce exposure to SHS. The purpose of this study was to determine SHS exposure at home and workplace, and its associated factors among non-smoker Malaysian adults.

**METHODS:**

Data were extracted from the Global Adult Tobacco Survey-Malaysia (GATS-M) that involved a representative sample of 5112 Malaysian adults. Logistic regression analyses were performed to examine the association between SHS exposure, sociodemographic factors, knowledge on the danger of SHS, and smoking restrictions at home and at work among non-smokers.

**RESULTS:**

Among non-smoker Malaysians, age ≥15 years, 27.9% (equivalent to approximately 4.21 million non-smokers) and 33.9% (equivalent to approximately 1.37 million non-smokers) reported that they were exposed to SHS at home and the workplace, at least once a month, respectively. Women (AOR=2.12, 95% CI: 1.61–2.78), young individuals (AOR=3.06, 95% CI: 1.48–6.33), Malays (AOR=2.39, 95% CI: 1.56–3.64) or other Bumiputra ethnic groups (AOR=2.40, 95% CI: 1.39–4.19) and those who worked as other than government employees were more likely to report SHS exposure at home (non-government employee: AOR=1.88, 95% CI: 1.06–3.36). Respondents with a total smoking restriction at home did not report any SHS exposure at home. Similarly, those whose workplace had smoking restrictions were less likely to report SHS exposure at the work compared to their counterparts whose workplace had partial (AOR=3.08, 95% CI: 1.84–5.15) or no smoking restrictions (AOR=15.33, 95% CI: 6.75–34.86).

**CONCLUSIONS:**

A substantial proportion of Malaysian adults were exposed to SHS at home and at work. The findings emphasize the need for policies on smoking restrictions at work and the need to promote the adoption of a completely smoke-free home, among the Malaysian population.

## INTRODUCTION

Secondhand smoke (SHS) contains at least 250 toxic chemicals, 50 of which have been identified as being carcinogenic^1,2^. Numerous epidemiological studies have shown that exposure to SHS increases the risk of lung cancer, coronary heart disease and stroke^3,4^. The first global study, in 192 countries, on the effects of SHS exposure revealed that about 0.6 million deaths and the loss of 10.9 million disability-adjusted life-years were due to SHS^5^. In Malaysia, the National Health and Morbidity Survey in 2006 reported that 21.5% of Malaysian adults, age ≥18 years, were exposed to SHS in any area within the last 7 days, and the prevalence was significantly higher among females, single adults, urban dwellers, Bumiputras, those with higher educational attainment, higher income and of younger age^6^.

In view of this, the Malaysian government, through the Ministry of Health, undertook several initiatives to reduce SHS exposure, including introduction of smoke-free legislation^7^, prohibition of smoking in indoor public areas^7^, encouraging multisectoral cooperation in educational campaigns on the health harms of SHS, incorporating the smoke-free element into community-based interventional programmes^8^ and advocating the blue-ribbon initiative for smoke-free areas at the workplace. A previous study demonstrated that SHS exposure was reduced significantly in public areas that had been gazetted as smoke-free areas^7^. Nonetheless, domestic residences and indoor working areas, where most people spent their time, remain beyond the purview of anti-smoking regulations^7^, and the prevalence of SHS exposure there has yet to be investigated in Malaysia.

On the other hand, studies have revealed that SHS prevalence at home and in indoor working areas is higher among groups with lower income and education levels^9,10^, blue-collar workers, individuals with no restrictions against smoking at home or in working areas^10,11^, and those with less knowledge regarding smoking harms^10^. Although research has shown the dangers of SHS exposure at home and at the workplace, most of the studies were from developed or high-income countries. Thus, the findings may not be applicable to Malaysia, in view of cultural and gender dynamic differences between Malaysia and those countries^12^.

Therefore, the aim of the present study was to investigate the prevalence of SHS exposure at home and in indoor working areas, as well as its association with sociodemographic factors, knowledge of the hazards of smoking, and smoking bans at home and workplace, among non-smoker adults in Malaysia. The findings are expected to guide the development of intervention strategies to decrease SHS exposure, introduce policy, and implement suitable anti-smoking initiatives for the home and indoor working areas.

## METHODS

We conducted the Global Adult Tobacco Survey-Malaysia (GATS-M), which was a cross-sectional study with three-stage proportionate-to-size sampling method to select a representative sample of Malaysians age ≥15 years. Our definition of tobacco includes: manufactured cigarettes, hand rolled cigarettes, kreteks, tobacco filled pipe, curut, cigar or cigagrillo, sidha/hookah, and bidis.

The first strata consisted of 15 States in Malaysia, and the second strata were the divisions of urban and rural areas by State. The primary sampling unit was enumeration blocks (EBs), which are artificial geographical areas created by the Department of Statistics consisting of 80–120 living quarters (LQs), based on the 2010 population census. LQs were the secondary sampling unit. At the third stage sampling, one household member, age 15 years and older, from the selected LQs was selected via a simple random sampling method. A total of 426 EBs (222 urban and 204 rural) and 5112 LQs were randomly selected.

Face-to-face interviews were conducted by trained research assistants (RA) to obtain data from the selected respondents. The RA explained the purpose of the survey to the selected respondents. The interview session only commenced after written consent was given by the respondents. To ensure a high response rate, respondents who were away from home or from their LQs during the initial visit were re-visited up to three times. If the respondent was still unreachable after the third attempt, they were excluded from the study. The ethical clearance for the study was obtained from the Medical Research and Ethical Committee (MREC), Ministry of Health Malaysia.

The GATS-M questionnaire was adapted from the English version of the Global Tobacco Survey questionnaire, developed by the Center for Disease Control and Prevention and the World Health Organization. The questionnaire was translated, and pre-tested among 120 respondents to establish face validity of the instrument. Minor modification of the questionnaire was carried out based on the responses during the pretest.

Only non-smokers (respondents who answered ‘no’ to the item: ‘Do you currently smoke tobacco?’) were included in the analysis. The dependent variables were ‘exposed to SHS at home’ and ‘exposed to SHS at work’. Respondents who reported that someone smoked in their home at least once a month were categorized as being exposed to SHS at home. Those who reported working while someone smoked in the building of their workplace, at least once a month, were classified as being exposed to SHS at work.

Independent variables were restrictions of smoking at home and at work (total restriction, partial restriction, no restriction). Restriction of smoking at home was measured by the item: ‘Which of the following best describes the regulation about smoking in your house?’, with the choices of ‘smoking is allowed in the house’, ‘smoking usually is not allowed but there are exceptions’, ‘not allowed in the house’ and ‘no regulation’. Those who answered ‘smoking is allowed in the house’ and ‘no regulation’ were classified as ‘no smoking ban at home’, whilst those who selected ‘smoking usually is not allowed but there are exceptions’ and ‘not allowed in the house’ were categorized as ‘partial restriction of smoking’ and ‘total restriction of smoking at home’, respectively. Smoking policy at work was examined by the item: ‘Which of the following best describes the smoking policy in the building at your workplace?’. The choices were that smoking is: ‘allowed everywhere’, ‘allowed in certain places’, ‘not allowed anywhere in the building’, and ‘no policy’. Respondents who said smoking was ‘allowed everywhere’ and ‘no policy’ in their workplace were categorised as ‘no smoking ban at working areas’. Those who responded ‘allowed in certain places’ were categorised as ‘partial restriction/ban at working areas’, whilst those who answered ‘not allowed anywhere in the building’ were considered as having ‘total smoking restriction/ban at working areas’. Knowledge of SHS was measured using four items: 1) SHS causes serious illness to a non-smoker?, 2) SHS causes heart diseases in adults?, 3) SHS causes lung cancer among adults?, and 4) SHS causes lung disease in children?; with responses ‘yes’ or ‘no’. Sociodemographic variables consisted of gender, ethnicity, education level, age group, residential areas and marital status. The socioeconomic status of the respondents was measured using the wealth index, constructed based on the information on household ownership of assets via principal component analysis. We then divided the summary score into wealth quintiles, with the lowest quintile (Q1) being the poorest and the highest (Q5) being the richest.

Prior to data analysis, thorough data cleaning was performed, and data were weighted according to sociodemographic attributes, such as gender, residential area (urban or rural) and four standard age groups based on the 2010 Malaysian population census. The sociodemographic attributes of respondents were portrayed using descriptive analyses while the proportion of respondents with SHS exposure at home or at work was presented via cross-tabulation. The associations between SHS exposure at home or workplace with independent variables (gender, age group, residential area, educational level, ethnicity, marital status, occupational status, income quintile, smoking bans at home or workplace, knowledge of SHS effects on health) were determined via multivariable logistic regression (MLR). The MLR was carried out by ‘Enter method’ to determine the ‘real effect’ of each independent variable with dependent variable, after controlling for the confounding effects of other independent variables. Two-way interactions were tested among the variables in the final model and no significant interactions were detected among them. All analyses were carried out using SPSS (Complex sample design) version 20 statistical software and results were presented with a 95% confidence interval (CI) and p-values for statistical significance.

## RESULTS

A total of 4250 respondents participated in the study, yielding a response rate of 83.1% (4250/5112). The majority were non-smokers (76.9%), with a significantly higher proportion among the women, among the elderly and young adults, with higher education levels, and of Chinese or Indian descent. However, no significance difference in the proportion of non-smokers was observed between residential areas (Table 1).

**Table 1:** Sociodemographic characteristics of non-smoker respondents, age ≥15 years, in Malaysia, with weighted percentages

*Demographic characteristics*	*n*	*N (thousands)*	*%^a^*	*95% CI^a^*	
				*Lower*	*Upper*
**Gender**					
Male	1144	5938	56.1	52.7	59.4
Female	2125	9887	98.7	98.0	99.1
**Age group (years)**					
15–24	605	4745	83.3	79.7	86.4
25–44	1284	6063	71.0	67.8	73.9
45–64	1026	3764	77.3	74.1	80.2
65+	354	1252	85.0	80.1	88.8
**Residence**					
Urban	1616	11485	77.3	74.6	79.8
Rural	1653	4340	75.7	73.3	78.0
**Education level**					
Less than primary	520	1605	80.3	75.8	84.1
Primary	834	3170	75.7	72.1	79.0
Secondary	1031	4770	74.9	71.9	77.6
College or above	264	1472	84.7	80.1	88.4
**Ethnicity**					
Malay	1931	9143	75.4	72.7	77.9
Chinese	553	3226	84.6	80.5	88.0
Indian	213	1552	80.4	73.6	85.8
Other	572	1903	70.0	64.7	74.9
**Marital status**					
Married	2094	9222	76.9	74.7	78.9
Single	754	5295	75.0	71.4	78.2
Divorced/separated/widowed	409	1145	87.9	83.8	91.0
**Occupation**					
Government employee	293	1336	73.9	68.4	78.7
Non-government employee	727	4317	65.6	61.9	69.2
Self-employed	470	1722	55.4	50.6	60.2
Unemployed	291	1140	94.9	93.2	96.1
Homemakers/students/retired	1468	7246	83.2	76.7	88.1
**Income Quintile^b^**					
Q5	698	4941	82.9	79.3	86.0
Q4	689	3832	80.8	76.9	84.2
Q3	601	3004	71.8	67.1	76.0
Q2	628	2281	73.0	68.1	77.5
Q1	603	1578	68.0	62.9	72.7

N – estimated population, n – sample.

a Weighted values based on estimated population.

b Quintile 5 is the most affluent group, while Q1 is the poorest group, according to the wealth index.

### Prevalence of SHS and factors associated with its exposure at home

Approximately one-quarter (27.9%; 95% CI: 25.5–30.4) of non-smoker Malaysians, age ≥15 years (equivalent to approximately 4.2 million people), reported that they were exposed to SHS at home at least monthly. The multivariable logistic regression showed that the prevalence of exposure to SHS at home was significantly higher among the women (AOR=2.12, 95% CI: 1.61–2.78), those 15–44 years old (with 15–24 years having AOR=3.06, 95% CI: 1.48–6.33; and 25–44 years an AOR=2.15, 95% CI: 1.19–3.89), of Malay (AOR=2.39, 95% CI: 1.56–3.64) or other indigenous descent (AOR=2.40, 95% CI: 1.39–4.19), of married status (AOR=2.08, 95% CI: 1.20–3.57) or single status (AOR=1.61, 95% CI: 1.01–2.63), who worked as other than government employees (non-government employee, AOR=1.88, 95% CI: 1.06–3.36; self-employed, AOR=2.22, 95% CI: 1.12–4.41; unemployed, AOR=3.23, 95% CI: 1.46–4.13), compared to their respective reference counterparts. In terms of smoking bans at home, respondents with a total smoking restriction at home reported no exposure to SHS within the past month, whilst respondents without a smoking restriction at home showed significantly higher prevalence of SHS exposure compared to those with a partial smoking restriction at home (Tables 2a and 2b).

**Table 2a:** Prevalence and factors associated with SHS exposure at home among non-smoker Malaysian adults age ≥15 years, percentages and adjusted odds ratios (AORs) are weighted

	*Exposure to SHS at home*					*Factor associated with SHS exposure at home*		

*Demographic characteristics*	*n*	*N (thousands)*	*%^a^*	*95% CI^a^*		*(AOR)^a^*	*95% CI^a^*	
				*Lower*	*Upper*		*Lower*	*Upper*
**Overall**	906	4217	27.9	25.5	30.4			
**Gender**								
Male	193	1095	19.5	16.2	23.0	Ref		
Female	713	3122	32.8	29.9	36.0	2.12*	1.61	2.78
**Age group (years)**								
15–24	201	1466	32.2	27.5	37.3	3.06*	1.48	6.33
25–44	360	1601	27.6	24.2	31.3	2.15*	1.19	3.89
45–64	264	891	24.8	21.0	29.0	1.66	0.93	2.60
65+	81	259	21.6	16.2	28.2	Ref		
**Residence**								
Urban	372	2745	25.1	22.1	28.3	Ref		
Rural	534	1472	35.0	31.5	38.0	0.87	0.64	1.18
**Education level**								
Less than primary	167	508	32.2	27.9	39.0	1.72	0.87	3.41
Primary	324	1352	29.5	25.7	33.6	1.57	0.88	2.81
Secondary	355	1949	28.5	25.4	31.9	1.21	0.71	2.05
College or above	59	405	18.9	13.9	25.0	Ref		
**Ethnicity**								
Malay	605	294	33.8	30.7	37.1	2.39*	1.56	3.64
Chinese	84	455	14.5	10.8	19.2	Ref		
Indian	25	215	14.4	9.2	21.8	0.91	0.45	1.85
Other	192	604	33.5	27.7	39.9	2.40*	1.39	4.19
**Marital status**								
Married	617	2482	27.9	23.2	30.9	2.08*	1.20	3.57
Single	203	1462	28.5	24.4	33.1	1.61*	1.01	2.63
Divorced/separated/widowed	85	267	24.2	18.3	31.3	Ref		
**Occupation**								
Government employee	41	208	16.3	11.5	22.4	Ref		
Non-government employee	169	1066	25.9	21.7	30.7	1.88*	1.06	3.36
Self-employed	113	396	24.0	18.9	30.0	2.22*	1.12	4.41
Unemployed	506	2197	31.2	28.1	34.5	3.23*	1.46	7.14
Homemakers/students/retired	76	346	33.1	25.1	42.1	2.25*	1.24	4.13
**Income Quintile^b^**								
Q5	147	1087	22.8	18.9	27.3	Ref		
Q4	174	949	26.0	22.1	30.3	0.83	0.56	1.22
Q3	170	885	30.3	25.3	35.8	0.89	0.56	1.43
Q2	200	698	31.8	27.2	36.8	0.96	0.59	1.56
Q1	201	541	37.9	32.6	43.5	0.80	0.48	1.34
**Smoking ban at home**								
Yes^c^			-^c^				-^c^	
Partial	118	756	41.8	34.9	49.0	Ref		
No	780	3428	57.6	53.8	61.2	15.87*	12.58	19.65

a Weighted values based on estimated population.

b Quintile 5 is the most affluent group, while Q1 is the poorest group, according to the wealth index.

c For smoking ban at home = Yes, no one in this level reported SHS exposure at home.

Design effect of SHS exposure at home — 2.49.

* Significant at p<0.05.

**Table 2b:** Prevalence and factors (knowledge of health effect of SHS) associated with SHS exposure at home among non-smoker Malaysian adults age ≥15 years, percentages and adjusted odds ratios (AORs) are weighted

	*Exposure to SHS at home*					*Factor associated with SHS exposure at home*		

*Variable*	*n*	*N (thousands)*	*%^a^*	*95% CI^a^*		*(AOR)^a^*	*95% CI^a^*	

				*Lower*	*Upper*		*Lower*	*Upper*
**Knowledge of SHS effects on health**								
SHS causes serious illness to nonsmokers								
Yes	760	3601	27.0	24.8	29.3	Ref		
No	146	616	34.3	27.9	41.3	1.47	0.97	2.22
SHS causes heart disease in adults								
Yes	714	3450	28.5	26.1	31.0	Ref		
No	192	717	25.3	21.2	29.9	0.88	0.56	1.37
SHS causes lung cancer in adults								
Yes	774	3733	28.2	25.6	30.9	Ref		
No	131	471	25.0	20.1	30.8	0.76	0.43	1.35
SHS causes lung diseases in children								
Yes	777	3748	28.2	23.6	30.9	Ref		
No	129	469	25.6	20.2	31.9	0.85	0.52	1.41

a Weighted values based on estimated population.

Design effect of SHS exposure at home — 2.49.

### Prevalence of SHS and factors associated with its exposure at work

Tables 3a and 3b show that about a third (33.9%; 95% CI: 29.5–38.6) of Malaysians who worked indoors were exposed to SHS. Exposure to SHS was significantly higher in working areas with partial (AOR=3.08, 95% CI: 1.84–5.15) and no smoking restriction (AOR=15.33, 95% CI: 6.75–34.86), compared to their counterparts whose workplace had implemented smoking bans. Other sociodemographic factors such as gender, age, ethnicity, residential area, educational level, marital status, occupation, income level and knowledge of the health hazards of SHS were not associated with SHS exposure at the workplace (Tables 3a and 3b).

**Table 3a:** Prevalence and factors associated with SHS exposure at the workplace among non-smoker Malaysian adults age ≥15 years, percentages and adjusted odds ratios (AORs) are weighted

	*Exposure to SHS at work*					*Factor associated with SHS exposure at work*		

*Sociodemographic characteristics*	*n*	*N (thousands)*	*%^a^*	*95% CI^a^*		*(AOR)^a^*	*95% CI^a^*	

				*Lower*	*Upper*		*Lower*	*Upper*
**Overall**	239	1373	33.9	29.5	38.6			
**Gender**								
Male	114	705	39.1	32.6	46.0	1.22	0.80	1.90
Female	125	668	29.8	24.7	36.1	Ref		
**Age group (years)**								
15–24	36	320	30.6	21.6	42.3	Ref		
25–44	137	770	34.5	28.9	40.5	1.91	0.34	10.34
45–64	60	259	35.6	27.3	44.8	2.27	0.40	12.84
65+	6	24	53.2	18.7	84.9	2.89	0.08	18.18
**Residence**								
Urban	160	1141	35.6	30.4	41.3	1.52	0.89	2.56
Rural	79	233	27.4	20.9	35.0	Ref		
Education level*								
Lower than primary	11	25	49.8	29.0	70.6	1.11	0.32	3.78
Primary	54	289	47.5	38.2	56.9	0.91	0.48	1.72
Secondary	116	657	30.2	24.2	37.0	0.71	0.42	1.21
College or above	58	402	33.5	26.0	41.8	Ref		
**Ethnicity**								
Malay	145	753	30.7	25.0	37.0	Ref		
Chinese	52	367	41.2	32.8	50.0	1.02	0.53	1.95
Indian	23	161	36.8	24.8	50.7	1.31	0.55	3.13
Other	19	91	34.7	21.7	50.3	0.82	0.34	1.99
**Marital status**								
Married	159	858	35.3	30.4	40.5	1.35	0.69	2.63
Single	63	450	30.8	22.8	40.3	Ref		
Divorced/separated/widowed	17	64	40.4	23.7	59.7	1.44	0.29	7.10
**Occupation**								
Government employee	59	268	27.5	20.4	35.9	Ref		
Non-government employee	134	846	34.6	28.4	41.3	0.99	0.56	1.74
Self-employed	42	221	46.5	34.5	58.9	1.23	0.53	2.83
**Income Quintile^b^**								
Q5	64	430	29.2	22.8	36.5	Ref		
Q4	67	440	41.6	32.9	51.0	1.76	0.94	3.29
Q3	46	234	28.6	20.1	39.1	1.02	0.51	2.01
Q2	31	110	26.2	16.6	35.5	0.59	0.25	1.36
Q1	28	137	53.2	35.7	73.3	2.66	0.87	8.13
Smoking ban at working areas								
Yes	92	539	21.5	17.1	26.7	Ref		
Partial	82	478	46.1	37.3	55.2	3.08*	1.84	5.15
No	61	320	79.2	65.8	88.2	15.33*	6.75	34.86

a Weighted values based on estimated population.

b Quintile 5 is the most affluent group, while Q1 is the poorest group, according to the wealth index.

Design effect of SHS exposure at work place — 1.58.

* Significant at p<0.05.

**Table 3b:** Prevalence and factors (knowledge of health effect of SHS) associated with SHS exposure at the workplace among non-smoker Malaysian adults age ≥15 years, percentages and adjusted odds ratios (AORs) are weighted

	Exposure to SHS at work					Factor associated with SHS exposure at work		

Variable	n	N (thousands)	%^a^	95% CI^a^		(AOR)^a^	95% CI^a^	

				Lower	Upper		Lower	Upper
**Knowledge of SHS on health**								
SHS causes serious diseases to non-smokers								
Yes	213	1235	34.2	29.4	39.3	Ref		
No	19	267	33.0	14.7	58.6	0.78	0.16	3.90
SHS causes heart disease in adults								
Yes	194	1135	33.7	28.9	38.9	Ref		
No	45	238	35.1	24.8	47.0	1.36	0.47	3.90
SHS causes lung cancer in adults								
Yes	210	1215	33.0	28.5	38.0	Ref		
No	28	145	41.0	27.6	55.9	1.91	0.20	17.86
SHS causes lung diseases in children								
Yes	210	1206	33.1	28.6	38.0	Ref		
No	29	167	41.5	23.7	56.9	1.27	0.11	11.73

a Weighted values based on estimated population.

Design effect for SHS exposure at work place — 1.58. * Significant at p<0.05.

## DISCUSSION

This study revealed that the proportion of non-smokers in Malaysia (27.9%) who were exposed to SHS at home was comparable to that in Brazil (27.9%)^13^ and Ukraine (23.5%)^13^ but lower than that reported^14^ in China (67.3%), Philippines (44.8%), Bangladesh (43%) and Thailand (39.1%). On the other hand, the proportion of non-smokers that were exposed to SHS at their workplace was similar to that reported in the third (33%, year 2008) and fourth waves (29.9%, year 2009) of the International Tobacco Control survey in Malaysia^14^, as well as that reported in Poland (33.9%)^15^, but lower than that reported in Bangladesh (54.6%)^13^. These findings suggest that the SHS exposure in the workplace leveled off over the last 2 years in Malaysia^14^, and compared to other low- and middle-income countries, the level of SHS exposure among Malaysian non-smokers was moderate^13^. The disparity in SHS exposure between countries was probably due to the differences in social approval of smoking and the comprehensiveness of tobacco control measures implemented in the different countries. Nonetheless, future studies should include an item on the smoke-free gazettement status of the workplace, in order to scrutinize the actual association between smoke-free regulation and SHS exposure.

### Factors associated with SHS exposure at home

The present results show that female respondents are more likely to be exposed to SHS at home, which is in line with the findings reported in Bangladesh^10^ and Thailand^16^. However, Kaleta et al.^15^ reported no such association among Polish adults. The gender differences in SHS exposure between Asian and Western countries^10,15,16^ may be attributable to cultural differences. Due to the patriarchal structure of Asian societies, including Malaysia, where men are dominant over women, the women are less likely to express disapproval of the husband’s smoking behavior at home in order to maintain familial harmony^17^. In addition, the high prevalence of smoking among Malaysian men could also increase the likelihood of SHS exposure at home among their non-smoking spouses. This explanation is supported by the present findings that married non-smoker adults were more likely to be exposed to SHS at home than single or widowed/separated adults.

In the present study, those of younger age were more likely to be exposed to SHS at home, and this finding is in agreement with studies among non-smoking adults in Bangladesh^10^ and Thailand^16^. One plausible reason could be that young adults (15–24 years) were more likely to be economically dependent on their family and hence more likely to live with them. In hierarchical societies, like Malaysia, where the young have restricted autonomy, the sense of respect of the young for their elders discourages them from complaining about the smoking at home by the elders.

Compared to their counterparts of Malay or other descent, Malaysian Chinese had a lower prevalence of SHS exposure at home and this could probably be due to the lower prevalence of smoking among the Chinese. Furthermore, such low prevalence of smoking may potentially imply negative attitudes towards smoking and hence reinforce the norm of ‘say no to smoking at home’ among their family members, relatives or friends who smoke. On the other hand, the study showed that the likelihood of SHS exposure among government employees at home is significantly lower than that among their counterparts who are self-employed, working in the private sector, or homemakers. This could be due to the anti-smoking regulations^7^ implemented in government workplaces since 1993. Consequently, a norm of not smoking at the workplace may have been accepted by employees who then practiced it also at home.

In contrast to the findings of several studies performed locally and abroad^18,19^, the present study did not observe a significant association between knowledge of SHS health harms and SHS exposure at home. This finding suggests that knowledge of the effects of SHS on health is not sufficient to influence SHS exposure at home, and also implies that although some of them were aware of the risks of SHS exposure, they still avoided undertaking active and determined action to prevent SHS exposure. Similarly, education level was also found to be not associated with SHS exposure. This finding did not concur with the results of local and overseas surveys^18,19^ and refuted the notion that those with more education and better knowledge of SHS would avoid SHS exposure at home. One of the plausible explanations for such an observation could be that knowledge of the adverse effects of SHS did not motivate change, as the negative impact of SHS on health can become apparent after 20 years. This explanation is corroborated by the human behavior theory that posits that only imminent effects motivate changes in behavior^20^.

Despite the fact that the proportion of total smoke-free homes in Malaysia was only half of that reported in the USA (81.1%)^21^, none of the people who had implemented a total smoking restriction at home reported SHS exposure in the past month. The present findings are partly in line with those reported in another study^22^, where the SHS exposure among those who did not allow smoking at home was significantly lower compared to their counterparts who allowed it. These observations suggest that a total smoke-free home policy is needed to decrease SHS exposure at home.

### Factors associated with SHS exposure at work

The present study did not find a significant association between SHS exposure at the workplace and gender. These finding is not congruent with those reported amongst workers in Bangladesh^9^ and in the USA^23^. The present study did not find significant associations between ethnic groups and SHS exposure in the workplace, which implies that SHS exposure is similar across ethnicity, despite a generally lower prevalence of smoking among the Chinese and Indians. These observations can be explained by the fact that in the Asian setting, smoking is viewed as a moderator facilitating social communication^24^. Since a substantial portion of Malaysian Chinese and Indians (who are also more likely to be non-smokers) are involved in the business and service sector, which often requires socializing with business partners and colleagues, they avoid offending their customers by not disapproving their smoking at the workplace or advising them to smoke elsewhere.

Occupation (government employee, private, self-employed) showed no significant associations in both univariate and multivariate analyses in this study. Such findings are alarming and suggest violation of smoke-free laws among the government employees or visitors, as the SHS exposure among the government employees should be significantly lower in view of the legislation of smoke-free government facilities since 1993^7^. Therefore, enhanced enforcement by officers other than Environmental Health Officers from the Ministry of Health is warranted.

Respondents with partial or total smoking restrictions at work had a significantly lower likelihood of SHS exposure in the workplace. This finding was consistent with those of other cross-sectional^25^ and longitudinal^26^ studies, and can be explained by Siegwart Lindenberg goal framing theory^27^. The theory suggests that individuals tend to follow the regulations at their place of work to avoid embarrassment, in front of their superior or peers, for not following the rules or regulations stipulated in their workplace, and also because of the need to belong to the group and be similar to the others.

### Strengths and limitations

The selected sample in this study was representative of Malaysian adult population, age 15 years and older, which with the high response rate (81.5%) enables generalization of the findings to the Malaysian population. Data were collected by trained interviewers who followed the standard operating procedure of written interviewer, which might reduce systematic bias. The personalized approach employed in this study could encourage greater willingness of participants to respond honestly about exposure to SHS. In addition, quality control measures implemented reduced errors during data collection.

However, there are several limitations in this study. Firstly, the cross-sectional design of the study limits causal inferences to be made about the findings. Secondly, SHS exposure was self-reported without objective measurement, such as cotinine in saliva, and hence subject to recall bias. Thirdly, this study did not investigate the dimensions of exposure, such as frequency and intensity. Although we recognize the limitations of the study in terms of exposure variable, and despite the nature of the survey and the method of self-reported SHS exposure without objective measurement, our approach still provides reliable information on SHS exposure among non-smokers in Malaysia.

## CONCLUSIONS

The GATS Malaysia 2011 revealed that a substantial proportion of Malaysian adults were exposed to secondhand smoke at home and the workplace, which is most likely due to tobacco control measures in Malaysia not prioritized yet for home and workplace. Thus, health promotional and interventional programs should be enhanced, by encouraging voluntary smoking bans at home.
